# Impact of fecal microbiota transplantation in dogs

**DOI:** 10.3389/fvets.2025.1505226

**Published:** 2025-07-01

**Authors:** Leon Cantas, Rasmus Goll, Christopher G. Fenton, Ruth H. Paulssen, Henning Sørum

**Affiliations:** ^1^PrivateVET Small Animal Clinic, Norwegian Private Veterinary Services, Hammerfest, Norway; ^2^Biocode Bank Norway, Hammerfest, Norway; ^3^Research Group of Gastroenterology and Nutrition, Department of Clinical Medicine, UiT-The Arctic University of Norway, Tromsø, Norway; ^4^Clinical Bioinformatics Research Group, Institute Clinical Medicine, UiT-The Arctic University of Norway, Tromsø, Norway; ^5^Department of Paraclinical Sciences, Faculty of Veterinary Medicine, Norwegian University of Life Sciences, Ås, Norway

**Keywords:** dog, diarrhea, intestinal microbiota, transplantation, FMT

## Abstract

**Background:**

The digestive tract hosts a variety of microorganisms. These microorganisms “*micro-organs*” play multiple crucial roles in physiological, immunological, and metabolic processes in the body. The manipulation and transplantation of “*micro-organs*” have lately gained increasing interest in human medicine with promising clinical outcomes, whereas much less is known in veterinary practice.

**Objectives:**

The goals of this pilot study were to evaluate the safety and impact of Fecal Microbiota Transplantation (FMT) for dogs suffering from non-infectious digestive disorders.

**Animals:**

Seven client-owned adult dogs with idiopathic persistent diarrhea (>3 weeks) and very poor skin-coat conditions received the intervention (FMT) and were evaluated in a private veterinary clinic.

**Methods:**

Transplants have been taken from healthy donors and were administered rectally to recipients. Objective clinical examinations with analyses of blood and feces samples on day 0 (pre-FMT) and days 14–28 (post-FMT) were performed. Besides the conventional blood hematology and biochemistry analyses, 16S *rRNA* sequencing analysis was used in fecal samples.

**Results:**

No FMT-related complications occurred. Five of seven (71%) patients demonstrated improved fecal parameters associated with better overall clinical outcome, whereas four of the five (80%) recovered recipients showed molecular correlation with the donor gut microbiota after rectal FMT. There were insignificant changes shown for the conventionally analyzed blood samples. The serum cobalamin levels showed a tendency to increase in recovered recipients.

**Conclusion:**

FMT was easy to apply and displayed certain health benefits in this study. Our findings reveal the important role of a *“re-gained”* gut microbiome balance in the overall health of dogs. Further research is needed to identify the dynamics and interplay between the different bacterial phyla that may have an impact on the stimuli of host immunologic and metabolic responses.

## Introduction

The digestive tract of animals and humans contains a collection of various microorganisms, “*micro-organs*” (1), which live and *“function”* in a highly complex ecosystem (2, 3). The important role of microbiota changes in the digestive tract (dysbiosis) has been related to several acute and chronic diseases, such as non-infectious inflammatory enteropathies and inflammatory bowel disease (IBD), in dogs and cats, similarly to humans (4).

Recently, gut microbiome mapping tools have revealed that mammals host approximately ~ 10^13^ microbial cells, which is estimated to be 10 times greater than the number of body cells (5). The genomic content of these microbes is assumed to be over 100 times larger in amount than the human genome (6). Up to 98% of the gastric microbiota is dominated by *Helicobacter* spp. in cats and dogs, meanwhile, a less diverse microbiota has been found in the small intestine (mainly Firmicutes and Bacteriodes) compared to the large intestine (minimum 10 bacterial phyla) (7). Most of the physiologically active “*micro-organs*” live and function in the large intestine (1), which plays several important intestinal and extraintestinal roles in the following: (i) the fermentation process of non-digested substances (8, 9), (ii) production of several signal molecules (10), (iii) immune system cross-talk (11), and (iv) behavioral changes (12, 13).

Companion animal healthcare is one of the fastest-growing segments of the animal health industry after the global humanization of pets over the last decades. As for humans (14), non-infectious inflammatory enteropathies occur commonly across all veterinary settings despite improved pet hygiene (indoors) and selective breeding programs in developed countries (15). Several studies in humans and dogs have revealed that enteropathies are usually associated with alterations in the fecal microbial communities (4, 16, 17). Transplantation of the fecal “*micro-organs*” collected from clinically healthy donors to chronically diseased dogs is not commonly practiced in small veterinary clinics (18–20). We have therefore assessed the molecular changes in gastrointestinal flora after FMT transplantation in chronically diseased dogs, while the overall health index, including stool quality, skin-coat conditions, and hematology and biochemical blood parameters, were monitored simultaneously.

## Methods

### Study group

Treatment of our study group with Fecal Microbiota Transplantation (FMT) was conferred with the Norwegian Food Safety Authority. Seven client-owned adult dogs (recipients), male/female (5/2), were evaluated from October 2017 to November 2018 at the PrivateVET Small Animal Clinic located in Hammerfest, the northernmost city in Norway. All patients who met the selection criteria to participate in this research project as recipients were suffering from non-infectious, primary enteropathies that could be classified as idiopathic persistent diarrhea (Table 1). All recipients had presented poor skin-coat conditions for at least 3 months. Secondary enteropathies caused by abnormalities in pancreas, liver, or by any other internal dysfunctions were excluded. Historically, all recipients responded poorly to previous diet changes (hypoallergenic feed and commercial exclusion diet), and no significant remission was found to occur either after antimicrobial (amoxicillin and metronidazole) or anti-inflammatory treatments (prednisolone), including pre-or probiotic supplements. None of the recipients received antimicrobials for at least 4 weeks prior to sampling. The pet owners consented to participate in the study by signing an Informed Consent Form for Research.

**Table 1:** Individual data from recipients with (R1-7) chronic health problems and from donors with optimal health conditions prior to FMT.

Dog nr	Age (year)	Gender	Breed	Stool scale							Skin & coat quality^a^			
				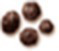	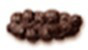	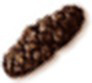	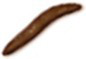	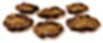	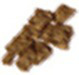	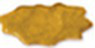	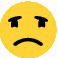 ←		→ 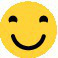	
				1	2	3	4	5	6	7	1	2	3	4
R1	11	F/N^b^	Jack Russel Terrier							X	X			
R2	7	F/N	Rottweiler						X		X			
R3	4	M^c^	Labrador							X	X			
R4	8	F/N	Bull terrier							X	X			
R5	7	F	Border collie						X			X		
R6	10	F	Golden retriever						X			X		
R7	9	M	Golden retriever							X	X			
Donor 1	4	F	Pointer			X								X
Donor 2	8	F	Alaskan husky			X								X
Donor 3	3	F	English setter			X								X

a Skin & coat quality: 1: poor, 2: good, 3: very good, 4: excellent.

b F/N: Female neutered.

c M: Male.

Four client-owned, physically and mentally robust, elite, and rewarded local dogs (donors) were included in this study. Donor exclusion criteria were as follows: (i) previous history of gastrointestinal skin problems, (ii) any other chronic physical and mental health problems, or (iii) ongoing use of medications, including previous antibiotic treatment during their lifetime. Donors were screened and found to have no carriage of parasites, bacterial enteropathogens (*Salmonella*, *Campylobacter*), extended-spectrum β-lactamase-producing organisms, or methicillin-resistant *Staphylococcus aureus.* Three of four potential donors were selected for harvesting of feces for further FMT. The fourth potential donor was excluded because of a subclinical *Giardia* spp. infestation.

### Evaluation of feces, skin, and coat quality

The consistency of the fecal samples was scored from 4 weeks pre-and 4 weeks post-FMT by using the 7-point Nestlé Purina fecal scoring system. These scores ranged from 1 (very hard, dry stools) to 7 (watery, no texture stools) (18). A modified clinical score-based approach was used to gain insight into a treatment activity index. The overall total score was determined to be either insignificant (0–3), mild (4–5), moderate (6–8), severe (9–11), and/or very severe (≥12) (21). The following criteria were scored from 0 to 3: activity, appetite, stool consistency and frequency, and skin-coat conditions. In addition, a sensory evaluation panel was used in this study to assess the impact of FMT on dogs’ skin and coat quality. Overall skin and coat quality were classified as either 1 (poor), 2 (good), 3 (very good), or 4 (excellent). Differences in gloss (visual light reflection from coat), optimal coat feel (by touching the coat’s softness without greasy or dry feel), and hair loss (assessing five areas by lifting the hair) were evaluated (22).

### Sample collection and processing

Fecal samples were harvested from donors and recipients (pre- and post-FMT) by collecting the samples at spontaneous defecation before feces hit the ground. Fecal samples were archived at −40°C after fecal flotation and Giardia testing (SNAP Giardia Test kit, IDEXX Laboratories, Westbrook, Maine, USA). Blood (5 mL) was evacuated in EDTA tubes and tubes without anticoagulants from *Vena jugularis externa/interna/communis* using 5 mL plain syringes with 23-G needles. The timing for blood and fecal sampling was pre- (day 0) and post-FMT (days 14 and 28). The differential blood cell counting went as follows: erythrocytes (ERY), hematocrit (HCT), hemoglobin (HEM), leucocytes (LEU), granulocytes (GRA), lymphocytes (LYM), monocytes (MON), and eosinophils (EOS) were measured (Vet abc Plus+, scil VET). Evaluation of various key biochemical markers, including aspartate aminotransferase (AST), alanine aminotransferase (ALT), total-protein (Tprot), albumin (Alb), and globulin (GLB), was determined on each blood sample by an external laboratory, including serum vitamin B12 (vitB12) analyses.

### Fecal microbiota transplantation (FMT)

A total of 90 g of the donor feces were diluted and mixed in 100 mL sterile 0.9% NaCl solution with 30 mL sterile 10% glycerol. The feces from different donors were not mixed together before the FMT. Each recipient received FMT only from one selected donor. The fresh final content was aspirated in 60 mL syringes after thawing and connected to an Enema kit (Enema Set, Plasti-Med Ltd.) for further rectal administration to patients (recipients). All transplants were frozen at −40°C and archived for less than 28 days prior to administration.

Recipients had been fasted for 8 h and taken out frequently for defecation prior to FMT. The recipients had gone through clinical examination a short while after normal bowel emptying. Then, 120 mL of the room-temperature contents was slowly deposited through the anus via Enema kits without any sedation. A water-based and sterile lubricant gel have been used (K-Y^®^ Lubricating Jelly) to the tip of the enema bulb prior to rectal administration of the FMT. With proper insertion technique, the tip of the enema bulb was gently inserted 3–5 cm in the rectum.

The patients’ pelvic cavities were raised up (~45^o^) gently from the table for 10 min for proper diffusion of the contents. To enhance adequate mucosal *“engrafment”* of *“micro-organs,”* recipients were kept under observation in a clean and pheromone-neutralised room for at least 30 min with the owner. The FMT was performed twice with a 14-day interval.

### DNA isolation

Bacterial DNA was isolated using the QIAamp Fast DNA Stool Mini Kit (Qiagen, Cat no:51604) and the QIAcube instrument (Qiagen) according to the manufacturer’s protocol. DNA quantity was assessed by using a Qubit 3 fluorometer (ThermoFisher Scientific, Wilmington, Delaware, USA). DNA samples were kept at −20°C until further use.

### Library preparation and next generation sequencing

Libraries were prepared with the Illumina demonstrated 16S *rRNA* protocol (Part#: 15044223), a method for preparing samples for sequencing the variable V3 and V4 regions of the 16S *rRNA* gene. The amount of input material was 12.5 ng of total DNA. The libraries were normalized to 4 nM and subsequently sequenced with a MiSeq instrument (Illumina, USA) according to the manufacturer’s instructions.

### Data analysis

Base calling and quality scoring were performed as a first step, including a quality check on the onboard computer of the MiSeq instrument. The software used to classify 16S *rDNA* sequences from samples was the USEARCH native Bayesian classifier.^1^ The 16S *rDNA* database used by the USEARCH software was the recommended RDP database.^2^ RDP provides quality-controlled, aligned, annotated bacterial and archaeal 16S *rRNA* gene sequences. Partial least square (PLS) distance (23), *p*-value adjustment methods (24), and correlation tables were performed with Bioconductor R.^3^

## Results

### Pre- and post-FMT comparison of the fecal scoring, skin-coat conditions, and clinical activity

**Figure 1:**
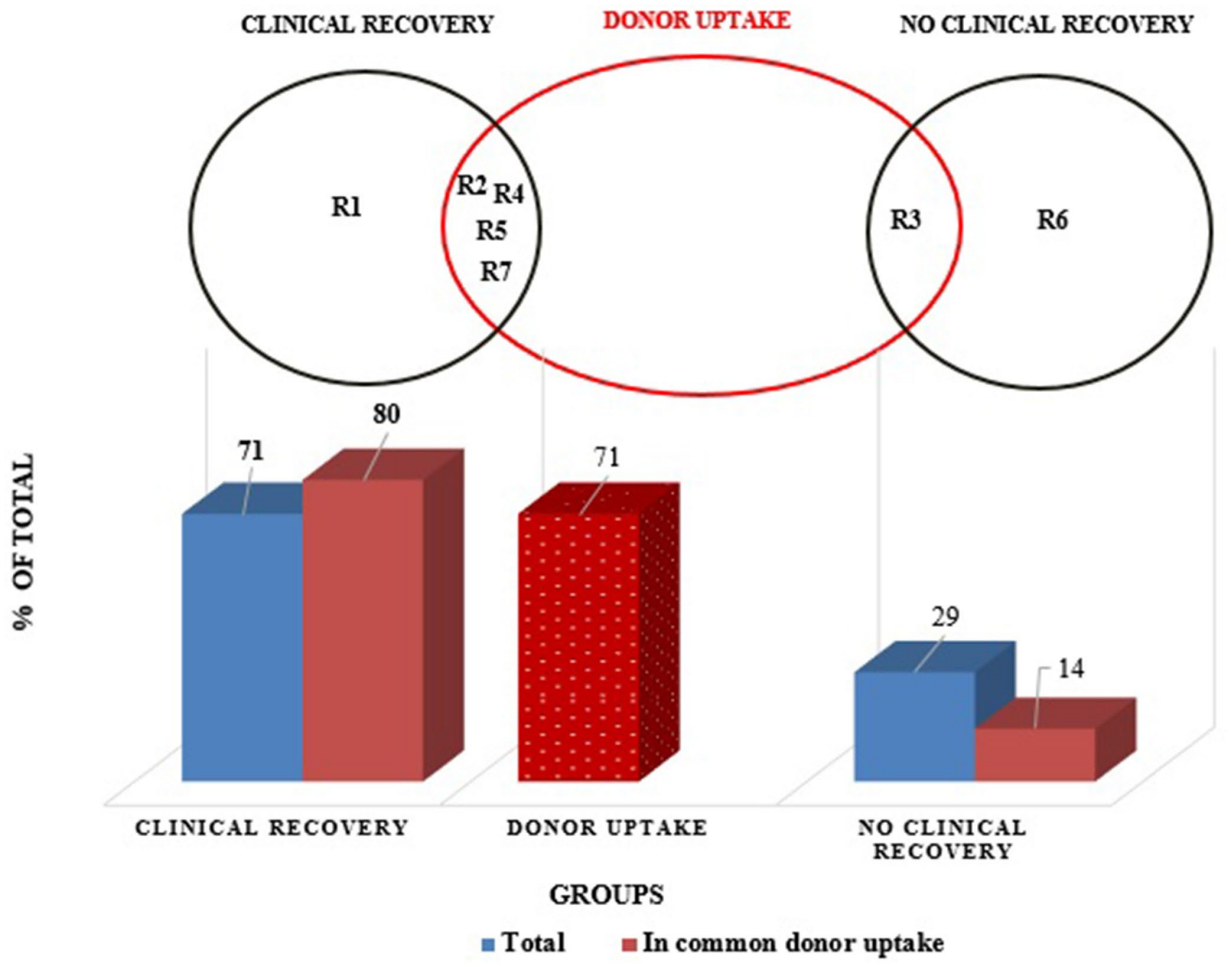
Distribution of study group (recipients) in clinically recovered and non-recovered groups related to donor uptake.

**Figure 2:**
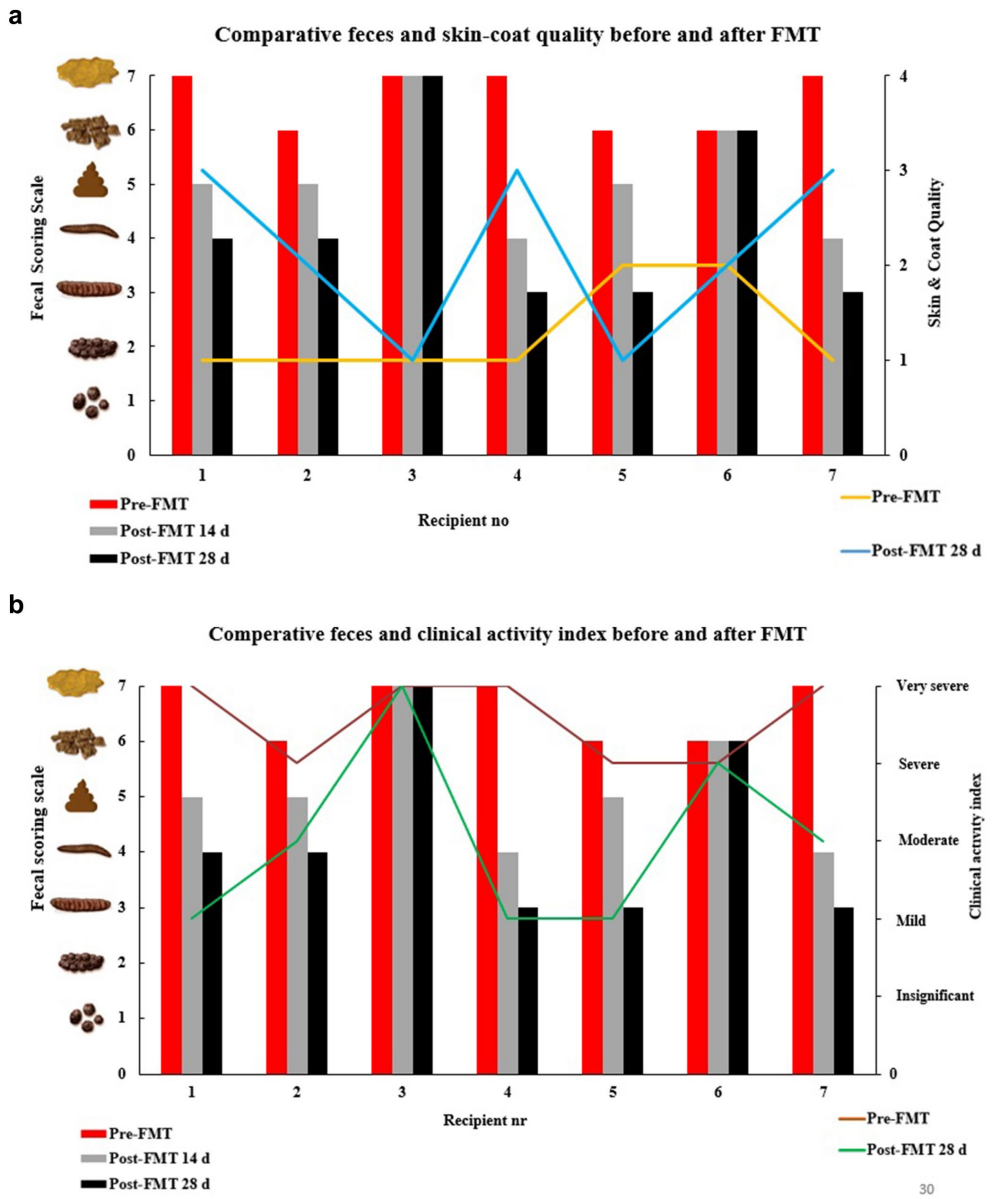
**(a)** Impact of FMT on fecal scoring scale and skin & coat quality in dogs. **(b)** Impact of FMT on fecal scoring scale and clinical activity index in dogs.

In total, five of seven (71%) recipients (R1,2,4,5, and 7) revealed an improved clinical activity index post-FMT, whereas recipients 3 and 6 showed no significant clinical improvement (Figure 1). None of the patients (recipients) got any worse fecal scoring, skin-coat quality, and overall clinical activity index after FMT treatments. R1, 4, and 7 presented with much better fecal scores (from quite watery stool without texture that was almost impossible to pick up to moisturized stool with distinct-log shape), including better skin-coat quality (from poor to very good condition with proved gloss, optimal coat feel and remarkable diminished hair loss) (Figures 2a,b).

### The gut microbiota composition of healthy donors

**Figure 3:**
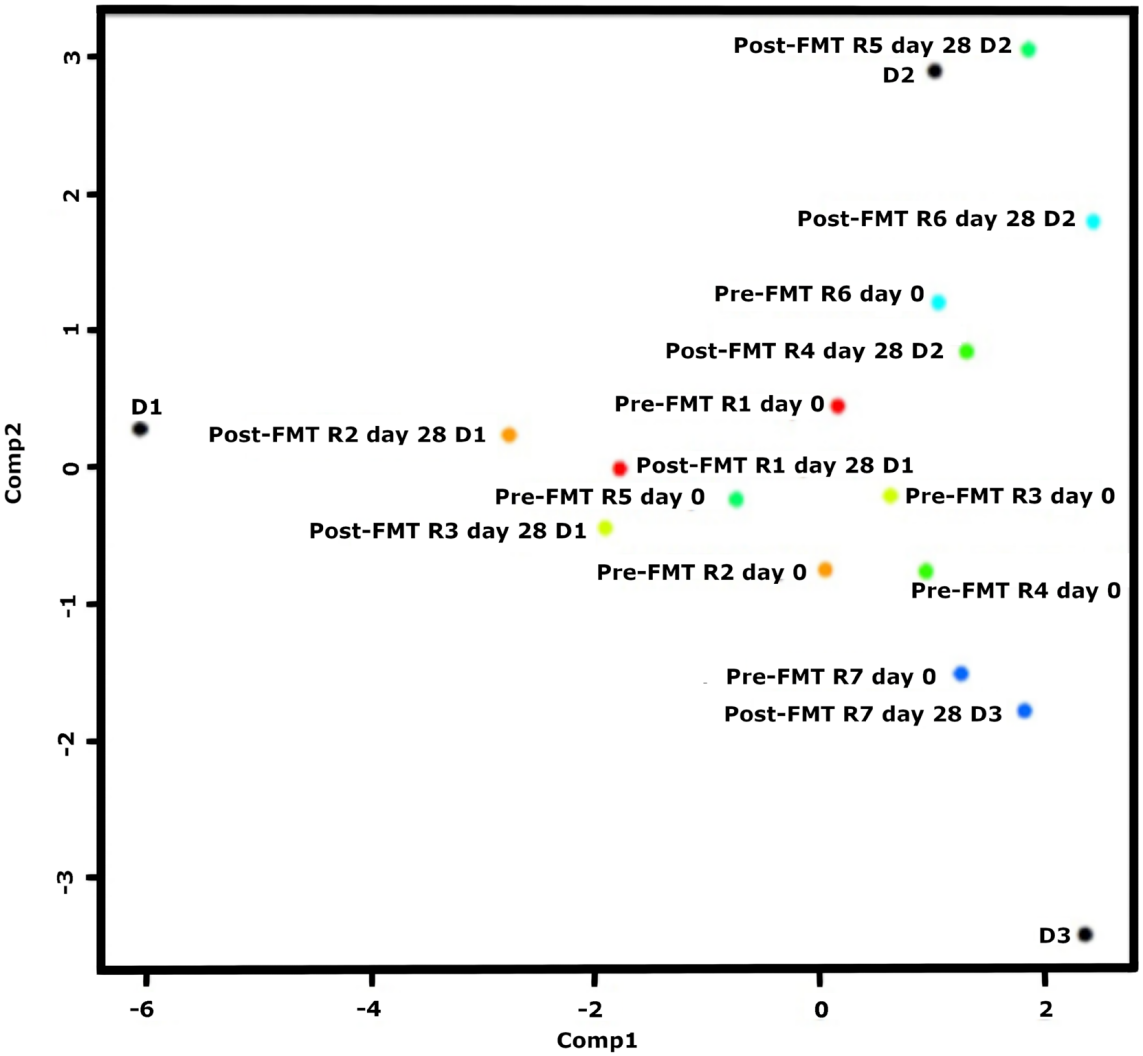
Recipients nr R2-3-4-5 and 7 move towards the donors, but RI and R6 seemingly did not get any effect from the donor. PLS similarity distance of D1 with R1 = 4.2, R2 = 3.2, R3 = 4.2, D2 with R4 = 2.06, R5 = 0.84, R6 = 8.6, D3 with R7 = 1.7.

The consistency of the fecal samples used for FMT from all donors was graded as 3 on the scale. The donors were presented with excellent skin-coat condition and an insignificant clinical activity index. *Clostridium* cluster XVIII (50%) and *Blautia* (18%) were the most abundant bacterial phyla in healthy donor 1 (D1), whereas *Clostridium* cluster XVIII (< 5%) and *Blautia* (10%) were found to be predominated by *Bacteriodes* (20%) and *Fusobacterium* (18%) in healthy donor 2 (D2). *Escherichia/Shigella* (~50%) have predominated over unassigned (10%), *Blatula* (5%), and *Bacteriodes* (5%) in the feces microbiome of donor 3 (D3). Overall, the proportionality of the microbiota composition was more homogenous, whereas several more bacterial groups were found in D2 compared to D1 and D3 (Figure 3).

### The gut microbiota pre−/post FMT

A variety of different bacterial groups were found among the recipients who suffered from almost similar idiopathic persistent diarrhea symptoms followed by poor skin-coat quality and very severe clinical activity index over several weeks. The composition of fecal microbiota in R1 as baseline revealed a closer relationship between R2, R6, and R7, whereas R3 was more comparable with R5 prior to FMTs. R4 was exceptional and contained larger microbial variation (Figure 3).

A clear donor–recipient molecular relationship was observed in four (80%) of the recovered FMT cases (R2, 4, 5, and 7), whilst no clinical activity index was recognized, even if a colonial donor–recipient relationship was found in R3 (Figure 3).

**Figure 4:**
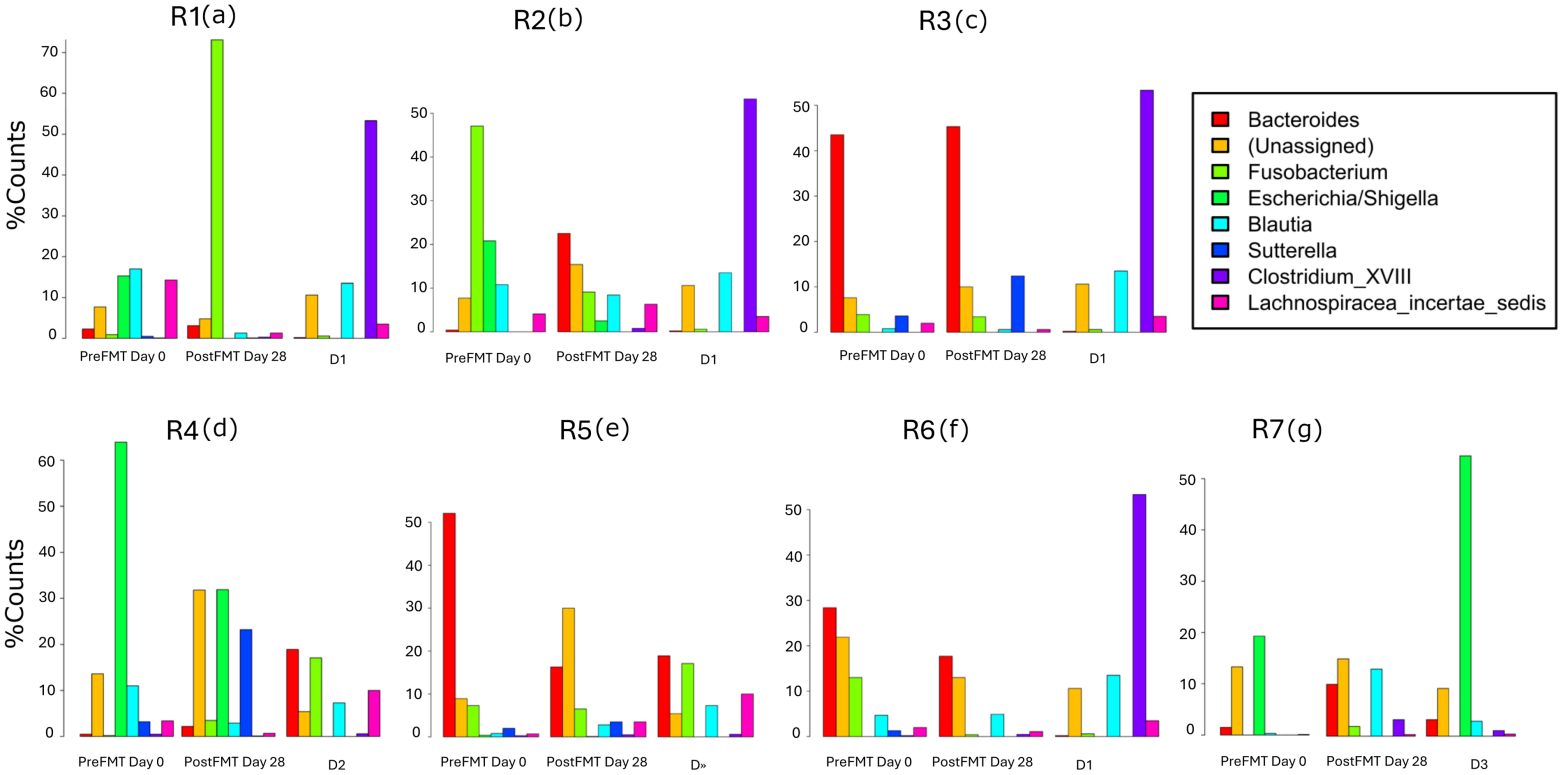
Post-FMT intestinal microbial community composition and structure changes in recipients (R1-7) **(a–g)** compared to pre-FMT and donors.

R1 showed a clinically remarkable response after FMT (donated from D1), but the bacterial communities were not proportional to the actual donor (*PLS =* 4.2) (Figure 4a). *Lachnospiracea incertae sedis* (7-fold), *Blautia* (9-fold), and *Escherichia/Shigella* (17-fold) were diminished, whereas *Fusobacterium* groups (35-fold) increased. None of the D1’s predominating *Clostridium* cluster XVIII were adopted in R1.

A ~5 fold decrease was found in *Fusobacterium* and *Escherichia/Shigella* phyla in R2 (*PLS =* 5.2) after FMT from D1 (Figure 4b), parallel to clinically improved skin-coat and digestive health (Figures 2a,b). On the other hand, *Clostridium* cluster XVIII and *Blautia* groups predominated fecal transplants from D1, and this resulted in neither clinical improvement nor deterioration in R3 (Figures 2a,b) despite the low PLS similarity distance (*PLS =* 4.2) of D1 (Figure 4c). Most of the fecal microbiota community remained unchanged while the *Sutterella* groups increased from 5 to 12% after FMT in this case.

The watery-mucoid diarrhea and poor skin-coat health conditions in R4 (*PLS* = 2.06) and R7 (*PLS =* 1.7) improved after treatment with transplants from D2 and D3, respectively (Figures 2a,b). Coincidentally, a 2-fold increase in unassigned bacteria phyla proportion was evident in R4 (Figure 4d) after FMT from D2, while *Blautia* increased over 10-fold in R7 after FMT from D3 (Figure 4g).

R6 showed no clinical improvement after FMT, donated from D2 (Figure 2b). The PLS similarity distance of D2 with R6 was found to be the highest (*PLS* = 8.6) in this study (Figure 4f).

### Evaluation of blood parameters in donors

**Figure 5:**
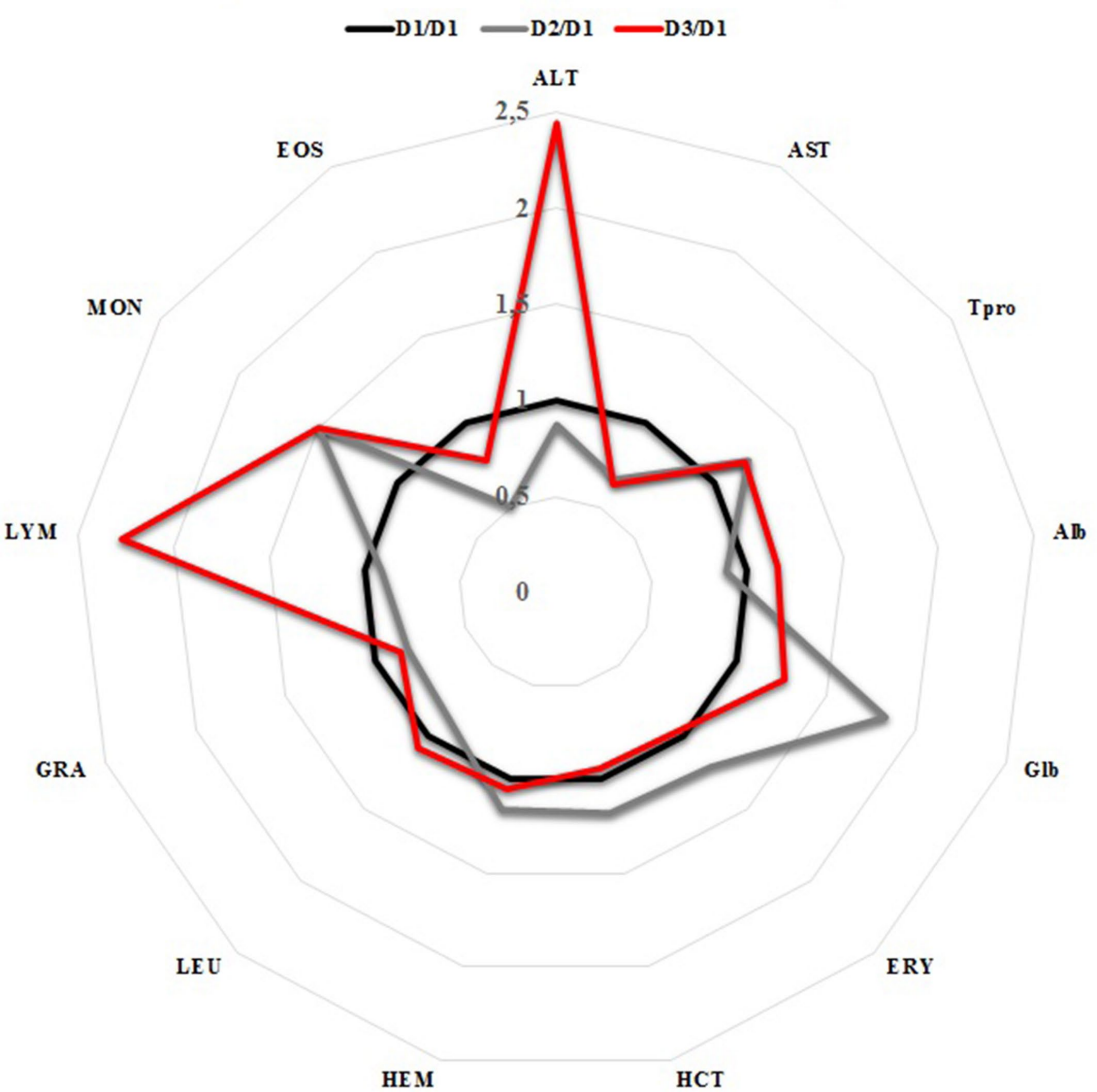
Relative comparison of the blood parameters among the FMT donors.

Analyzed biochemical and hematological parameters were within the reference ranges among the clinically health donors; however, D3 had slightly higher (~2 fold) LYM (2.5 × 10^3^/mm^3^, reference range 6.0–12.0×10^3^/mm^3^) and (~2 fold) ALT (53.6 U/L, reference range 10–118 U/L) compared to D1, without any clinical significance (Figure 5).

### Impact of FMT on blood parameters among the recipients

**Figure 6:**
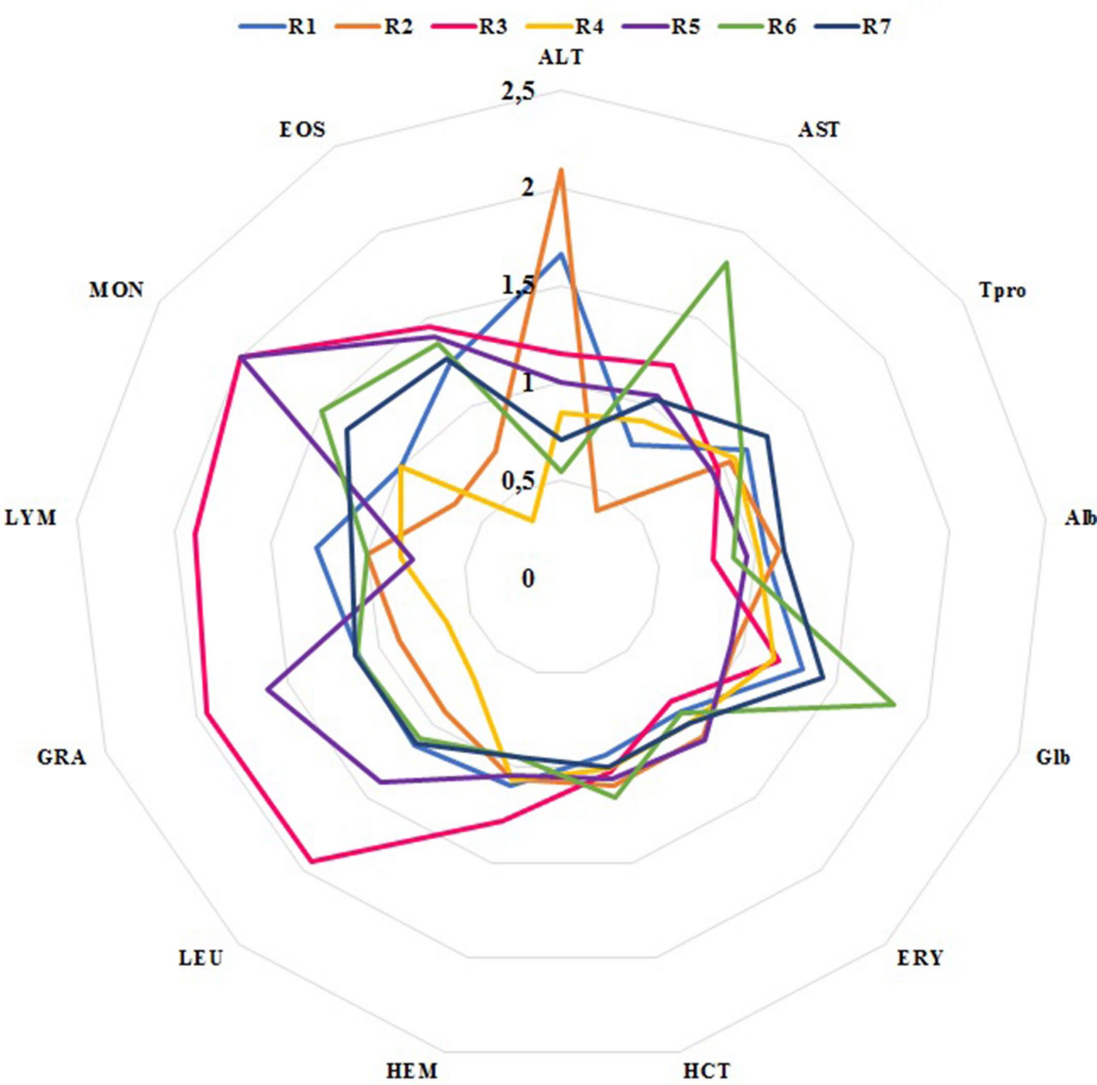
Relative blood parameter changes after FMT in recipients.

**Figure 7:**
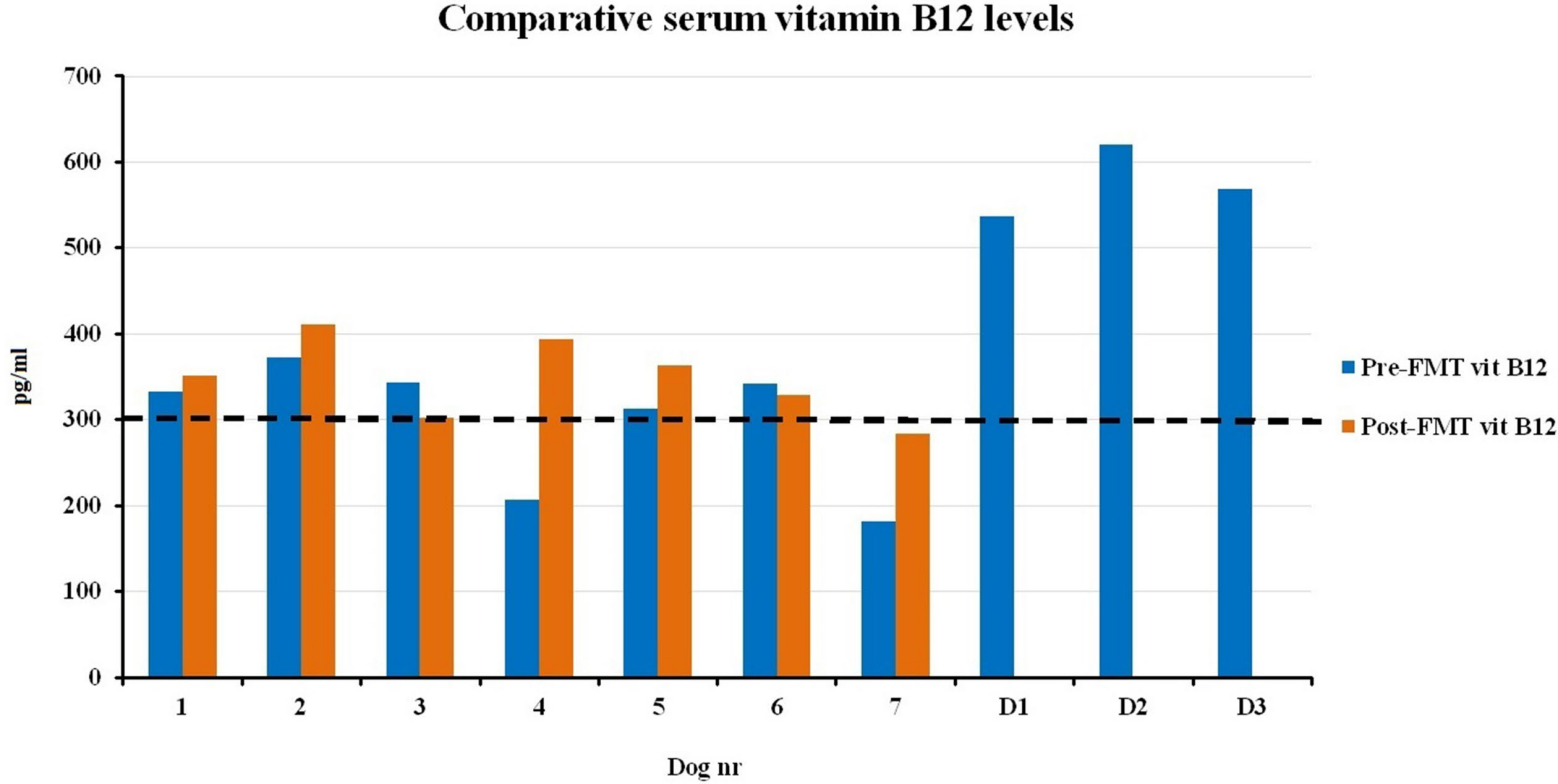
Comparison of serum cobalamin concentrations before and 28 days after FMT, including donors.

All recipients revealed slightly low count of ERY (mean ERY pre-FMT; 4.77 × 10^6^/mm^3^, reference range 5.50–8.50×10^6^/mm^3^), which remained stable after FMT (mean ERY post-FMT: 4.64 × 10^6^/mm^3^) (Figure 6). Even though the LEU remained nearly identical after FMT among the recipients, a slightly increased LEU (~2 fold) was found in R3 after FMT (9.5 × 10^3^/mm^3^, reference range 6.0–12.0 × 10^3^/mm^3^). Only R4 showed mildly elevated blood eosinophilia (0.64 × 10^3^/mm^3^, reference range 0.00-0.60 × 10^3^/mm^3^) values prior to FMT, whilst treatment showed a 3-fold reduction tendency in blood eosinophil cells. The presence of vitamin B12 deficiency improved on average (34%) in accordance with significantly improved fecal quality in R1, 4, 5, and R7; however, hypoproteinemia slightly increased in 14%. Otherwise, most of the other blood counts and serum biochemistry analyses apparently remained unchanged after FMTs (Figure 6). Relatively improved clinical outcome and skin-coat conditions were associated with elevated serum vitamin B12 levels in R4 and R7, though post-FMT 28d blood vitamin B12 levels were found to be barely above the reference range, at 326 and 316 (300–800 ng/ml), respectively (Figure 7).

## Discussion

In this study, we have for the first time employed a clinical FMT model in a Norwegian small animal clinic. A wide variety of chronic enteropathies with persistent and recurrent intestinal diseases are recommended to be treated with probiotic supplements, diet changes, immunosuppressants, and antimicrobials by the Norwegian antibiotic treatment guidelines for small animals (The Norwegian Medicines Agency, 2014). Treatment with FMT was thought to be a *“last cure option”* in this study since many other traditional (i.e., concurrent dietary changes, pre-and probiotics) and conventional (i.e., antimicrobials and immune suppressive) remedies have not improved the symptoms.

Information concerning the frequency and distribution of primary and secondary chronic canine bowel diseases is missing (15), but retrospective data from various developed countries pronounce such *“dysbiosis”*-related diseases as a growing and underestimated problem in small animal and public health (14, 15). Several studies in small animals and humans similarly have shown that the vast majority of acute and chronic diarrhea, as well as IBD, are related to changes in the gut microbes or disrupted homeostasis among “*micro-organs*” (4, 25). The composition of the canine gut microbiota and its changes with gastrointestinal diseases have been studied by both culture-dependent (26) and culture-independent methods (16, 17, 27). Advanced molecular methods showed a surprising co-evolution process in host immune and metabolic systems correlated with adaptation of “*micro-organs*” living in our guts over thousands of years (28, 29), parallel to the constant alteration of food-feeding habits, lifestyles, and treatment protocols (such as, antimicrobials). In a medium-sized canine’s lifetime, around 2–3 tons of feed pass through the digestive tract, which is mutually processed by commensal “*micro-organs*” living in their guts. Certain pathogens, chronic stress conditions, fast diet changes, and treatment with antimicrobials are well-known risk factors for disruption of homeostasis among intestinal “*micro-organs*.” However, treatment of dogs with disrupted *“micro-organ homeostasis”* is challenging due to incomplete understanding of the pathophysiological basis of these cases (4). Internationally, the treatment protocols are adopted from human medicine, which are usually based on dietary modifications, pre-and probiotics (30), immunosuppressive drugs (31), and antimicrobials (32).

We have long since learned that the use of antibiotics inevitably entails the occurrence of unintended modification of *“micro-organ homeostasis”* and more resistant strains of bacteria (34, 68). Globally increasing antimicrobial resistance is a great threat to modern veterinary and human medicine under the “One health concept” (33, 35, 36). Until recently, new antibiotics were repeatedly created to combat new strains of resistant bacteria. However, it is becoming increasingly obvious that this approach will not be possible in the long run because bacterial resistance is developing faster than the development of novel antibiotics (37). Norway has one of the lowest consumption of antimicrobials in animals, which reduces the selection of resistant bacterial strains, compared to the rest of Europe (38). Herein, alternative treatment approaches are needed to find ways to successfully diminish and secure the future use of antimicrobials in veterinary medicine for certain diseases (such as diarrhea and skin problems) that frequently occur due to disturbed *“micro-organ homeostasis”* or a malfunctioning metabolic and immune system.

Nutritional supplements like pre-and probiotics and their combination “*synbiotics*” have shown some clinical success (20, 39) in practice through *“smaller spectrum”* modification of the gut microbiota. On the other hand, *“broader–complete*” restoration of the healthy *“micro-organs”* has been achieved by FMTs (40). Severely damaged organs have been compensated for in patients (recipients) with the transplantation of *“organs”* from healthy donors in modern medicine. FMT has received considerable attention in human medicine with high restoration rates of “*micro-organ homeostasis”* in the past decade (41–43). On the other hand, relatively less attention has been paid in modern veterinary medicine to FMT (18, 19) even though “*old-school”* field veterinary surgeons have used transference of rumen microbes from healthy to sick cows as a rumination *“kick-start”* in the 1600s (Hieronymus Fabricius from Acquapendente). Currently, there is a lack of complementary scientific data regarding the optimal application of FMT in small animal patients (18, 44). The mechanisms behind certain health benefits are not well understood by the restoration or reconstruction of the gut microbiota. Nevertheless, faster resolution of clinical symptoms has been recently reported in dogs with significant changes in gut microbiota after FMT (20, 44, 45).

The *“optimal”* gut microbiome composition of the fecal *“micro-organ transplants”* has not yet been described in dogs; consequently, it was not possible to match the donors with the recipients. All healthy donors in this study delivered stool samples that contained diverse microbes in different proportions. We preferred to use FMT from a single donor, “*less is more-*FMT,” rather than mixing up the different fecal material from healthy donors, “*the more the merrier* FMT,” in this study. Future advanced metagenomic research could, for instance, investigate whether mixing different healthy FMTs may achieve better gut health and overall clinical wellness in small animals.

Clostridium cluster XVIII (50%) was found to be ~10-fold higher in D1 than in D2 and D3. The importance and reason for this bias are unknown. Inter-individual shift variations may exist (46, 47). The gut bacterial differences have been revealed from fecal samples of dogs with and without chronic intestinal inflammation. Several studies in small animals have shown a consequent reduction in *Firmicutes* and *Bacteriodes* phyla (25, 48). On the other hand, *Proteobacteria* and *Actinobacteria* phyla showed a tendency to increase in chronic bowel inflammation (48). None of the recipients had a clonal relationship with donors with regard to fecal abundance of bacterial groups before FMT in this study.

We were molecularly able to “*re-isolate*” the fecal microbiome of the donor for five out of seven (71%) recipients. Four of five (80%) clinically recovered patients showed obvious fecal colonial relationships to their donors that correlated with better skin-coat and clinical activity index. These encouraging results were in accordance with earlier reports: 100% recovery after FMT (45) and 79% recovery after FMT combined with standard treatment (20) in canine gastrointestinal patients. Only one case (R3) showed no significant improvement in symptoms despite engraftment of donor *“micro-organs.”* R6 is missing both clinical and molecular FMT effects in our study. The reasons for these biases are still unknown. A recent veterinary FMT study in horses has revealed almost no significant difference in intestinal microbiota. Meanwhile, treatment did not affect clinical survival and diarrhea control in recipient horses despite the high safety and simplicity of the FMT procedure (49). It is known that the canine intestinal tract is shorter and less complicated than the horse intestine.

This current study might have been improved if instrumental and invasive investigations like endoscopy-colonoscopy imaging followed by histopathological biopsy samples for advanced metagenomic analyses (bacterial and host immune system gene expressions) were applicable. But none of the clients who participated in this study were willing to perform such costly and challenging examinations. However, not all biopsy samples are equally interpretable in companion animals (50).

All blood samples from clinically healthy donors were found to be within the reference range, but D3 (English setter, a versatile hunting dog with excellent physical stamina) revealed slightly higher LYM and ALT (2-fold) compared to D1 and D2, without any clinical significance. Such transient increases in LYM and ALT (as much as three times normal values) can result from an acute surge of epinephrine, which can be released after prolonged excitement or exercise in high-performance dogs, such as sled dogs (51). In particular, mild anemia was common among our recipients before and after FMT. Low RBC may indicate chronic intestinal inflammation and hematochezia (none of the patients had fresh blood in feces) (52). There was a tendency towards increased total serum protein values without significant variations in the clinically recovered group after FMT. Lowered blood total protein levels among recipients prior to FMT were in accordance with previous reports, which may be due to increased protein leakage into the gut lumen or poor protein uptake (21, 53, 54). On the other hand, the actual reason for the eosinophil reduction in R4 remains unknown.

Vitamin B12 is a well-known unusual vitamin that is absent in plants but synthesized only by certain microorganisms in the gut, and it is associated with the balance between gram-positive and gram-negative bacteria (55–57). In line with other reports, serum vitamin B12 levels were found to be lowered in our recipients, which strongly indicates an existing intestinal malabsorption and IBD (53, 58). It has long been known that vitamin B12 deficiency may cause several clinical and metabolic problems like anemia, poor skin-coat condition, immunodeficiency, and changes in the digestive canal, including villous atrophy and malabsorption (59, 60). The treatment efficacy and cost of vitamin B12 deficiency (oral vs. parenteral administration) have been the topic of great debate among practitioners over the decades (61). The B12 values in blood showed a slight tendency to increase in completely recovered recipients (R4, R5, and R7) after FMT in this current study. The gram-negative balance was reduced by approximately 10% in R4 post-FMT day 28, despite the increased level of the *Pseudomonas* genus. None of our donors and patients received vitamin supplementation during this current FMT treatment trial. The actual reason for increased vitamin B12 biosynthesis among our recipients after FMT is unknown, but active involvement of the intestinal gut microbiome (i.e., *Propionibacterium shermanii* and *Pseudomonas denitrifican*) in the vitamin B12 synthesis process has been previously documented (62, 63). However, the potential constructive impact of vitamin B12 on human gut microbial communities has been previously postulated (55).

The safety of the FMT is not easy to determine in veterinary medicine, mainly due to breed variations and different screening procedures (19). Adverse effects were found to be quite uncommon in humans (64, 65). Previously reported side effects of FMT in humans are pathogen transmission or increased body weight (66). For these reasons, optimal donor selection plays a key role. Selection of vaccinated donors with excellent physical and mental health status (preferably, rewarded) and perfect body condition score prevents such potential side effects mentioned above in canine recipients. We took exclusion criteria a step further by eliminating donors that fit into the following criteria: (i) were exposed to any antimicrobials in the past and (ii) possessed any transmissible antibiotic resistance gene elements (such as; ESBL and MRSA). Antimicrobial therapy of FMT recipients who have received microbiota with antibiotic resistance genes may induce dissemination of resistance-carrying gene elements (i.e., plasmids). Thus, there is a great need for caution to improve the quality of fecal implants from donors to avoid further antimicrobial resistance genes acquisition and dissemination when carrying out FMT.

The Norwegian companion animal health care and welfare system is significantly ahead of the other European countries (Union of European Veterinary Practitioners, 2006), thanks to the well-functioning society and good prophylactic small animal services (from breeding to vaccination). The low transmissible disease prevalence and mobile antimicrobial resistance gene dissemination may count as an advantage with regard to great access to a larger potential donor pool in Norway than in the rest of Europe (Biocode Bank Norway / Leon Cantas, personal research notes, 2017–2018). Herein, screening and archiving of pet-specific auto-Fecal Microbiota Transplants (Biocode Bank Norway/Leon Cantas, personal research notes, 2017–2025) may evolve the future of FMT in predisposed individuals during their lifetime after, for example, prolonged antimicrobial therapy against persistent bacterial infections or use of chemotherapy to treat cancer in companion animals. In summary, veterinarians and small animal owners may start to look at healthy fecal matter as something more than waste: something that can be conserved and deposited at a *“local stool bank”* on good days for future bad days.

A most remarkable result of the current study was the correlation between increased fecal quality and improved clinical activity index as a result of FMT. Medicines that may give an additional boost and/or prolongation of the clinical recovery, such as multi-vitamin and symbiotic supplementation, may be worth administering to challenging cases after completing initial FMT (67) (Biocode Bank Norway/Leon Cantas, personal research notes, 2017–2025).

We think that the current results provide even more insight into the subtle and immediate effects of FMT, which is responsible for improved clinical outcomes. Introduction of FMT in veterinary practice to prevent and/or treat certain gastrointestinal and skin diseases can be recommended. Further studies are still needed to shed new light on the current findings and to clarify the underlying mechanisms.

There is a low correlation between the dominating bacterial phyla sequenced from feces and the clinical effect of the FMT. The reason for this is unknown. However, this observation may indicate that there are bacterial phyla with a small number of cells in the feces that may have a beneficial impact on the intestinal disorder and diarrhea.

## Conclusion

Chronic enteropathies have always been challenging to manage in small animal clinic practice. Treatment with FMT can be considered as an alternative if other diet changes and therapeutics fail to improve the symptoms. Undoubtedly, the use of feces as a part of the cure is not aesthetically superior to the other commercially used *“quick fix solutions”* in veterinary and human medicine. Nevertheless, small animal clinicians may, under controlled circumstances, stick to the adage “*Vets advise FMT – clients decide,*” which may improve symptoms in a cost-effective way.

## Data Availability

The datasets presented in this study can be found in online repositories. The names of the repository/repositories and accession number(s) can be found in the article/supplementary material.

## Footnotes

^1^ https://www.drive5.com/usearch/manual/exmiseq.html

^2^ https://sourceforge.net/projects/rdp-classifier/

^3^ https://www.bioconductor.org/
